# Abdominal Pain Followed by Altered Mental Status: A Rare Presentation of Guillain Barré Syndrome

**DOI:** 10.7759/cureus.27550

**Published:** 2022-08-01

**Authors:** Shekhar Shekhar, Avi Harisingani, Nikita Gupta

**Affiliations:** 1 General Medicine, Grant Government Medical College and Sir J.J. Group of Hospitals, Mumbai, IND

**Keywords:** syndrome of inappropriate antidiuretic hormone, acute abdominal pain, altered mental status, hyponatraemia, guillain barrè syndrome

## Abstract

Guillain Barré syndrome (GBS) is an acute inflammatory polyneuropathy with several variants; it usually presents as acute symmetrical muscle weakness with or without bulbar involvement, making it a neurological emergency. In this report, we describe the case of a 39-year-old male who presented with abdominal pain for three days and whose illness became progressively drowsy on the fifth day. Based on clinical assessment, nerve conduction studies, and biochemical, microbiological, and radiological investigations, other causes were ruled out and it was concluded that the patient had hyponatremia secondary to syndrome of inappropriate diuretic hormone secretion (SIADH) due to GBS.

Although sensory symptoms like pain or dysaesthesias occurring in the back or extremities are common and may precede motor weakness, abdominal pain remains a very rare presentation of GBS. GBS is usually understood as a “pure” peripheral nervous system disorder but central nervous system (CNS) dysfunction may occur due to metabolic abnormalities (like hyponatremia and CO_2_ narcosis) or autonomic dysfunction in GBS, its treatment, or due to GBS itself (Anti-GQ1b disease variant).

## Introduction

Guillain Barré syndrome (GBS) is a cause of acute weakness commonly following an infection. There are many variants of GBS, the most common being acute inflammatory demyelination polyneuropathy (AIDP). Other sensorimotor variants are acute motor axonal neuropathy (AMAN), acute motor sensory axonal neuropathy (AMSAN), pure sensory GBS, and Miller Fisher syndrome. MFS presents as ophthalmoplegia, ataxia, and areflexia but sometimes all the symptoms might not be present. Bickerstaff brainstem encephalitis is a type of MFS that includes ataxia, and ophthalmoplegia along with encephalopathy. Both MFS and Bickerstaff brainstem encephalitis have been associated with anti-GQ1b antibodies. Other rare variants include pharyngeal-cervical-brachial weakness, acute pandysautonomia (diarrhea, vomiting, abdominal pain, ileus, and urinary retention), acute bulbar palsy, facial diplegia, and distal limb paresthesia [1]. Our patient presented with abdominal pain followed by altered mental status. Back pain and lower limb pain are common in GBS whereas presentation of GBS with abdominal pain is extremely uncommon [2].

## Case presentation

The patient was a 39-year-old male, Indian Muslim, working as a taxi driver, who presented to the hospital with the chief complaint of abdominal pain for three days and was admitted under general surgery. He described stabbing, intermittent, non-radiating pain in the lower half of the abdomen (more so in the periumbilical region), lasting for minutes to hours, severe enough to wake him up at night, relieved on walking/moving about, without diarrhea, constipation, or vomiting. The patient had not had any fever or gastrointestinal or urogenital symptoms prior to this illness. On examination, he was vitally stable with tenderness in the periumbilical region on deep palpation with no guarding. A digital rectal examination was normal. There was no history of significant weight loss. He did not consume alcohol, tobacco, or recreational drugs. Complete blood count, blood sugar, erythrocyte sedimentation rate (ESR), and C-reactive protein (CRP) were within normal limits. Serum sodium and potassium were 137 meq/L and 4.2 meq/L respectively. Renal function and liver function tests were within normal limits. Stool chemical examination was negative for occult blood. Stool microscopy did not show any parasitic forms, pus cells, red blood cells, eosinophils, or fat globules. Fecal calprotectin and elastase levels were within normal limits. Stool cultures sent on two subsequent days were negative. Ultrasonography of the abdomen did not reveal any abnormality. The patient was given inj. pantoprazole 40 mg I.V. OD, inj. Drotaverine 40 mg I.V. BD, and inj. DNS (5% dextrose + 0.9% normal saline) 500 cc I.V. O.D. during his stay at the general surgery. He had not been taking any medications prior to the admission and denied any comorbidities. On day five of the illness and since the onset of abdominal pain (that is, day three of admission), the patient became drowsy (Glasgow Coma Scale score: 10, E2V3M5) and was brought under the care of medicine. On examination, he was afebrile, had no rashes, had a blood pressure of 130/80 mmHg, pulse rate of 84 beats/minute, regular, normal volume without any radio-radial or radio-femoral delay, and all peripheral pulses were felt equally; he had a respiratory rate of 16 cycles/minute, maintaining saturation on room air. He was drowsy with the eyes opening to painful stimuli and localizing to deep pain stimulus; the tone was decreased in bilateral lower limbs with absent ankle, knee, and plantar reflexes. The tone was also decreased in the upper limbs and biceps and triceps reflex, with absent supinator reflex. Power could not be tested as the patient was not following verbal commands, but lower limbs were observed lying helplessly and the patient localizing to deep pain stimulus with the upper limb.

Laboratory investigations revealed serum sodium of 118 meq/L (normal range: 135-145 meq/L), with normal serum potassium, renal function tests, and liver function tests. Arterial blood gas (ABG) was normal. Serum osmolality was 245 mOsm/kg (normal range: 285-295 mOsm/L). Simultaneously, urine osmolality was reported as 546 mOsm/L and urine sodium was 62 meq/L. The patient was clinically euvolemic with normal serum thyroid-stimulating hormone (TSH) and cortisol levels. Decreased serum osmolality with a urine osmolality of more than 100 mOsm/L, and urine sodium of more than 40 meq/L with no history of diuretic use were suggestive of the syndrome of inappropriate diuretic hormone secretion (SIADH). He was treated as a case of chronic (since the exact onset could not be determined), severe, euvolemic hyponatremia secondary to SIADH (cause under evaluation) with the administration of 3% hypertonic saline initially, followed by fluid restriction (<1000 mL/day). Sodium values repeated at 12, 24, 36, and 48 hours were 122, 124, 126, and 130 meq/L respectively. Contrast-enhanced CT brain did not reveal any abnormality. Urine porphobilinogen level was within normal limits. The patient regained consciousness with improving sodium levels and reported an inability to move his lower limbs, inability to turn on the bed, and difficulty in raising his arms above his head and gripping objects. He also complained of tingling in both lower limbs. He did not complain of having difficulty in speaking and swallowing. He did not have any bowel/bladder-related complaints. He had not been taking any drugs prior to the illness and his relatives denied any seizure-like activity. There was no history of similar self-resolving episodes or heavy metal exposure.

Neurological examination was consistent with a Modified Medical Research Council (mMRC) scale 0 power at bilateral hip flexors and extensors, knee flexors, and extensors and ankle dorsi- and plantar flexors with no flickering of toes. Bilateral knee, ankle, and plantar reflexes were absent. He had proximal (more than distal) weakness in upper limbs with mMRC scale 2 power on shoulder flexors and extensors, and 3 on the elbow, wrist flexors, and extensors. Biceps and triceps reflexes were present only on reinforcement. Sensory examination was normal in both upper and lower limbs. Although he did not complain of any difficulty in speaking or swallowing at that time, cranial nerve examination revealed bilaterally symmetrical lower motor neuron type facial palsy. Examination of rest cranial nerves was normal. There was no neck rigidity, and the examination of the spine was normal. He was clinically diagnosed as having GBS. CSF study was done on day seven of illness (that is day five of admission). CSF was clear, colorless, and CSF protein levels were 118 mg/dL (normal level: <45 mg/dL); CSF sugar level was 80 mg/dL, with 2/mm^3^ mononuclear cells and no RBC on microscopy, suggestive of albumino-cytological dissociation. Nerve conduction study (NCS) was suggestive of acute motor axonal neuropathy (AMAN variant of GBS) affecting lower limbs more than upper limbs with normal sensory NCS. Since GBS was suspected, stool antigen test and PCR for campylobacter were done, which returned negative. Even though intravenous immunoglobulin (IVIG) was initiated, the patient developed quadriplegia, bulbar weakness, and respiratory muscle weakness requiring mechanical ventilation. Unfortunately, he succumbed to ventilator-associated pneumonia.

## Discussion

Our patient presented with abdominal pain followed by altered mental status. Stool routine examination, microscopy, and cultures obtained on two subsequent days were negative for any infective etiology. Complete blood count, ESR, and CRP were within normal limits. Ultrasonography of the abdomen was normal. Metabolic causes of abdominal pain with neurological symptoms like diabetic ketoacidosis, uremia, and porphyria were ruled out as blood sugar, renal function test, and urine porphobilinogen were normal. Other causes of abdominal pain with neurological symptoms [3] like nutritional deficiencies (thiamine, niacin, Vitamin E), inflammatory bowel disease, celiac disease, and Whipple's disease were highly unlikely given the short clinical history. The patient did not have any history of exposure or examination findings suggestive of lead or arsenic toxicity. Altered sensorium was attributed to hyponatremia due to SIADH. Once sodium was corrected and the patient became oriented, his neurological symptoms were investigated, and GBS was found to be the underlying cause of his abdominal pain, hyponatremia, and weakness (that occurred last in this case).

One-third to two-thirds of patients with GBS experience pain in the acute phase, more frequently in the lower back and lower limbs. The mechanism of pain can be multifactorial. Firstly, inflammation of large myelinated nerve fibers can cause muscle pain in limbs with dysesthesia. Secondly, nerve root pain can cause radicular pain shooting into the limbs. Thirdly, small fiber involvement can also cause pain in GBS [4]. The presentation of GBS with abdominal pain is extremely uncommon. Abdominal pain can be due to sensory nerve inflammation, dorsal nerve root inflammation, or gastrointestinal autonomic dysfunction [2]. Since other autonomic symptoms and signs were not observed in our patient, it was unlikely that autonomic dysfunction was the cause of the abdominal pain.

Central nervous system (CNS) involvement is classically excluded from the spectrum of GBS. However, it is not uncommon for GBS to present with an altered mental state [5]. CNS dysfunction in GBS correlates with more severe disease and the chance of requiring assisted ventilation [6]. Altered mental status in GBS may be due to any of the following factors: neuropsychiatric manifestations of the disease itself, metabolic and autonomic derangements caused by the disease, treatment of the disease, or GQ1b-related disease variants.

Neuropsychiatric manifestations of GBS include vivid dreams, hallucinations, illusions, and delusions - mostly of the paranoid type. These have been found to occur in GBS patients even prior to ICU admission (and hence differing from ICU delirium) in a prospective controlled study [7].

Among metabolic derangements, hyponatremia due to SIADH is common in GBS and develops in roughly half of the patients. This is due to altered hypothalamic osmoreceptors and ADH sensitivity of the renal tubules, both of which could arise from immune-mediated damage [8]. CO_2_ narcosis resulting from respiratory paralysis may also cause altered mental status.

Autonomic dysfunction is a characteristic feature of GBS and is seen in about two-thirds of all cases. This can cause CNS dysfunction by blood pressure fluctuation [5]. For example, severe hypertension can lead to posterior reversible encephalopathy syndrome (PRES), a condition that can result in seizures, altered mental status, and visual impairment. Orthostatic hypotension may lead to syncope.

Altered mental status can also result from the treatment of GBS. IVIG, a treatment for GBS, can cause adverse effects like thrombosis and aseptic meningitis. IVIG can also contribute to the development of PRES. Therefore, IVIG can also be a cause of CNS manifestations in GBS by these mechanisms [4]. It is important to note that IVIG causes pseudohyponatremia as serum osmolality remains normal.

Lastly, the GQ1b-related disease variant, namely Bickerstaff brainstem encephalitis, a condition closely related to the MFS variant of GBS, is a possible cause of altered mental state [9].

## Conclusions

GBS should be considered in differentials of acute abdomen with neurological symptoms after excluding other possible causes (depending on specific case scenarios) like infective ones (enteric fever, Lyme disease), metabolic disorders (diabetic ketoacidosis, diabetic neuropathy, uremia, porphyria), heavy metal intoxication (lead, arsenic), gastrointestinal disorders (celiac disease, Whipple's disease), and nutritional deficiencies (thiamine, niacin, vitamin E). Mental status abnormalities should prompt a search for alternative explanations but cannot rule out GBS.
